# Suppressed Ion Migration by Heterojunction Layer for Stable Wide-Bandgap Perovskite and Tandem Photovoltaics

**DOI:** 10.3390/molecules29174030

**Published:** 2024-08-26

**Authors:** Taoran Wang, Weiwei Zhang, Wenjuan Yang, Zeyi Yu, Gu Xu, Fan Xu

**Affiliations:** 1 Shenzhen Institute for Advanced Study, University of Electronic Science and Technology of China, Longhua District, Shenzhen 518110, China; yangw48@mcmaster.ca (W.Y.); xugu@mcmaster.ca (G.X.); 2 Department of Materials Science & Engineering, University of Toronto, Wallberg Memorial Bldg., 184 College St., Toronto, ON M5S 3E4, Canada; ww.zhang@mail.utoronto.ca; 3 China National Offshore Oil Corporation Huizhou Petrochemical Co., Ltd., Huizhou 516086, China; yuzy4@cnooc.com.cn; 4 Department of Materials Science and Engineering, McMaster University, 1280 Main Street West, Hamilton, ON L8S 4L8, Canada; 5 State Key Laboratory for Artificial Microstructure and Mesoscopic Physics, School of Physics, Peking University, Beijing 100871, China; 6 Frontiers Science Center for Nano-Optoelectronics & Collaborative Innovation Center of Quantum Matter, Peking University, Beijing 100871, China

**Keywords:** wide-bandgap perovskite, tandem solar cell, ion migration, heterojunction, simulation

## Abstract

Wide-bandgap (WBG) perovskite has demonstrated great potential in perovskite-based tandem solar cells. The power conversion efficiency (PCE) of such devices has surpassed 34%, signifying a new era for renewable energy development. However, the ion migration reduces the stability and hinders the commercialization, which is yet to be resolved despite many attempts. A big step forward has now been achieved by the simulation method. The detailed thermodynamics and kinetics of the migration process have been revealed for the first time. The stability has been enhanced by more than 100% via the heterojunction layer on top of the WBG perovskite film, which provided extra bonding for kinetic protection. Hopefully, these discoveries will open a new gate for WBG perovskite research and accelerate the application of perovskite-based tandem solar cells.

## 1. Introduction

Halide perovskite materials have attracted great attention recently due to their excellent optoelectronic properties, such as adjustable bandgap, large absorption coefficient, long carrier diffusion length, and high carrier mobility [1,2,3]. The certified power conversion efficiency (PCE) of perovskite solar cells (PSCs) has reached 26.1%, showing great potential to compete with silicon (Si)-based photovoltaic cells [4]. In general, perovskite exhibits a chemical formula of ABX_3_, where A stands for organic cations such as methylammonium (MA^+^) and formamidinium (FA^+^), B represents metal divalent cations (e.g., Pb^2+^ and Sn^2+^), and X represents halide ions including I^−^, Br^−^ and Cl^−^. By doping in the ABX positions, the bandgap of perovskite materials could be expanded to above 1.65 eV [5]. The resultant wide-bandgap (WBG) perovskites can be applied in multijunction tandem solar cells, whose maximum PCE has surpassed 34%, already breaking the Shockley–Queisser limit in single-junction solar cells [6,7,8].

However, WBG PSCs showed long-term instability issues, impeding their practical applications [5,9,10,11,12]. Unlike conventional silicon (Si), the perovskite structure is supported by weaker bonds, such as hydrogen bonding and Van der Waals bonding, which are vulnerable to moisture attack [13,14,15,16]. Additionally, the perovskite has a soft lattice, making the ions inside the structure easy to migrate, which inevitably leads to material decomposition, component loss, lattice collapse, phase transition, and ultimately the loss of photovoltaic properties [9,17,18]. As a result, the device lifetime of perovskite-Si tandem cells is mainly limited by the top WBG PSCs, rather than the bottom Si cells.

Although the influence of moisture attack can be reduced by proper encapsulation, degradation caused by ion migration remains, especially for WBG perovskites applied in tandem cells, as their lattices are heavily doped, causing many more defects [19,20,21,22]. Heat and light can easily activate ion migration in the WBG perovskite lattice, resulting in the deformation of the local crystal structure, which will further damage the perovskite film [23]. Some researchers have tried to apply halide salt to passivate the surface defects, which enhances the PSC’s stability as a result of the additional halogen bonding by the extra halide salt [24,25]. Unfortunately, the additional ions will aggravate ion migration and the lifespan can only be increased to about 2000 h, far from enough for commercialization [26]. On the other hand, others proposed to apply high-valent cation for interstitial doping to enhance the Coulomb attraction [27,28,29]. However, interstitial doping is not only more prone to diffusion, but also causes more lattice distortion, leading to strain-induced phase transition, which is detrimental to efficiency [28,29]. It is evident that it is unlikely that there is an effective technique with which to inhibit the ion migration by the current doping, encapsulation, and passivation method, especially when the detailed migration kinetics and thermodynamics remain unknown.

It is therefore the purpose of the current research to unravel the detailed migration kinetics and thermodynamics by simulation method. Moreover, the previous literature reports that WBG perovskites suffer severe ion migration issues [5], yet lacks related experimental methods to unravel the specific process. Although others have tried to use electron microscopy (e.g., SEM and TEM) and X-ray diffractions (XRD) to characterize the migration, these methods could only reveal specific moments, instead of the continuous migration process that has been long understood for decades [30,31]. By combining the bonding energy of the perovskite structure, the driving force of ion migration, and environmental interference factors, we successfully used Python to reveal the entire process of ion migration in a 100 × 100 lattice. It has been discovered that, unlike the conventional diffusion process, which generally diffuses from a certain source, ion migration may start anywhere inside the film due to the presence of imperfections in polycrystalline films. In addition, we also found that although extra halide doping is detrimental to stability, it will cause a rise in the migration activation barrier due to the extra halogen bonding, meaning the stability can be increased by the proper amount of doping. Moreover, the migration rate can be reduced by more than fourfold when applying an extra layer of stable perovskite to form a heterojunction. Hopefully, these discoveries can provide fresh inspiration for WBG PSC device fabrication technologies, and resolve one of the most significant challenges for their future commercialization.

## 2. Results and Discussion

**Figure 1:**
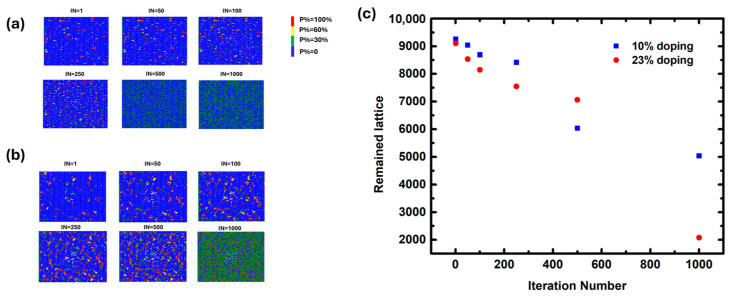
(**a**) The simulation results of ion migration at room temperature in perovskite film of 10% doping in the X site after iteration 1, 50, 100, 250, 500, and 1000. Blue = P: 0; green = P: 0–0.3; red = P: 0.3–0.6; and yellow = P: 1.0; the iteration number represents the migration time. (**b**) The simulation results of ion migration at room temperature in perovskite film of 23% doping in the X site after iteration 1, 50, 100, 250, 500, and 1000. (**c**) The scatterplot of the number of the remaining lattices after iteration 1, 50, 100, 250, 500, and 1000 for both doping proportions.

To simulate the ion migration in WBG perovskite, 10% doping in the X site was first simulated. As can be seen in Figure 1a, the number of remaining perovskite lattices was 9257 after the first iteration, which gradually decayed to 9040, 8691, 8417, 6036, and 5036 after the 50th, 100th, 250th, 500th, and 1000th iteration, respectively. The migration was initiated from the left lattice of the perovskite film; as this was the outer layer, part of the perovskite structure was unbonded, representing the lowest bonding energy. In addition, migration could also be triggered inside the film where imperfections were present, such as defects or grain boundaries. After 1000 iterations, the ion concentration will be homogenized, and the migration will stop. These discoveries are different from the previously uncovered moisture diffusion results, in which case an almost infinite amount of water molecules will diffuse into perovskite film from the environment, leading to an accelerated diffusion process instead of the current reduced one.

Next, the doping proportion was increased to 23% for the WBG simulation to represent the perovskite materials applied for tandem solar cells. As indicated in Figure 1b, the number of remaining perovskite lattices was 9106 after the first iteration, which gradually decayed to 8535, 8147, 7548, 7063, and 2080 after the 50th, 100th, 250th, 500th, and 1000th iteration, respectively. The degradation trend for this high concentration of doping was obviously different from that of low concentration, especially at iteration 500, after which a dramatic rise in the degradation rate was observed (Figure 1c). This was probably due to the extra halide ions providing more halogen bonding against the migration. As can be evidenced by the results in the low-concentration simulation, the dramatic rise in the degradation rate occurred at iteration 250. These unconventional results demonstrated that the migration was different from Fickion diffusion; the higher concentration gradient may not necessarily indicate a greater migration driving force, due to the presence of extra bonding, which means the ion migration can be inhibited by introducing extra halide ions.

**Figure 2:**
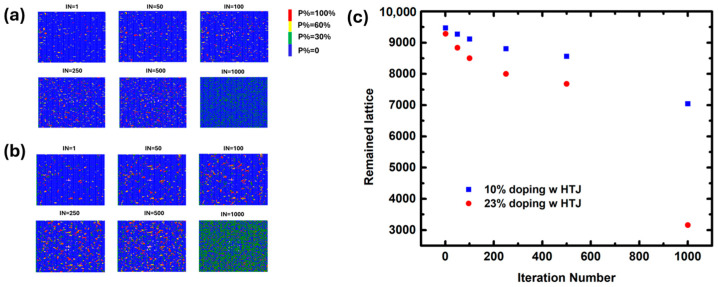
(**a**) The simulation results of ion migration at room temperature when the heterojunction layer is applied on top of the perovskite film of 10% doping in the X site after iteration 1, 50, 100, 250, 500, and 1000. Blue = P: 0; green = P: 0–0.3; red = P: 0.3–0.6; and yellow = P: 1.0; the iteration number represents the migration time. (**b**) The simulation results of ion migration at room temperature when the heterojunction layer is applied on top of the perovskite film of 23% doping in the X site after iteration 1, 50, 100, 250, 500, and 1000. (**c**) The scatterplot of the number of the remaining lattices after iteration 1, 50, 100, 250, 500, and 1000 for both doping proportions.

**Figure 3:**
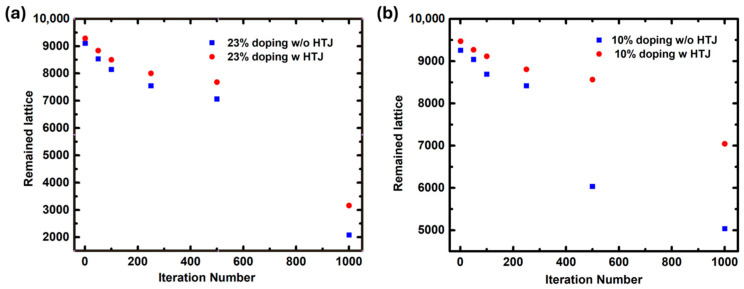
(**a**) The scatterplot of the remaining lattices in the perovskite film of 23% doping in the X site with and without the heterojunction layer. (**b**) The scatterplot of the remaining lattice in the perovskite film of 10% doping in the X site with and without the heterojunction layer.

Inspired by the halogen bonding, the effect of heterojunction was also simulated. The presence of a heterojunction layer on top of the perovskite film could provide extra bonding to the exposed perovskite lattice site. Thus, an extra bonding energy of 0.1–0.4 eV was applied to the model. As can be seen in Figure 2a, when the heterojunction layer was applied to the 10% doping perovskite film, the number of remaining perovskite lattices was 9427 after the first iteration, which gradually decayed to 9270, 9115, 8806, 8564, and 7045 after the 50th, 100th, 250th, 500th, and 1000th iteration, respectively. The number of remaining lattices for the 23% doping perovskite film was 9286, 8837, 8502, 8002, 7681, and 3162, respectively (Figure 2b). The overall degradation status is summarized in Figure 2c. Compared to their counterparts in Figure 1, the degradation rate is obviously reduced by at least 100%, as shown in Figure 3, proving that the extra bonding by the heterojunction layer is beneficial to the structural stability.

In order to obtain detailed kinetic information, the curve of the remaining lattices was plotted. As can be seen in Figure 3, the decaying trend in all conditions is similar, especially in the beginning stage, which demonstrated an exponential decline. Based on the Arrhenius term in kinetics, the exponential trend is directly related to the activation energy. Thus, it is evident that the perovskite with the heterojunction layer exhibited larger activation energy, proving that there is extra bonding between the heterojunction layer and the perovskite. In the latter stage of the migration, a sudden rise in the decay rate was observed; this was possibly due to the breakdown of the perovskite structure, which destroys the halogen and hydrogen bonding that hinders migration. It can be seen from Figure 3b that the accelerated decay is retarded when the doping proportion is small under the influence of the heterojunction layer, demonstrating the importance of kinetic protection.

**Figure 4:**
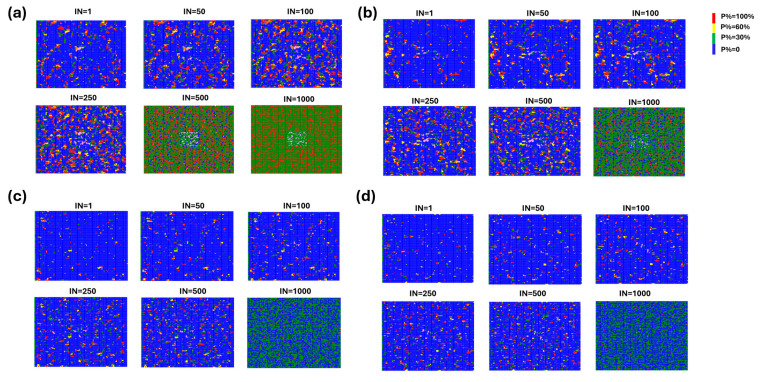
(**a**) The simulation results of ion migration at higher temperatures in the perovskite film of 23% doping in the X site after iterations 1, 50, 100, 250, 500, and 1000. Blue = P: 0; green = P: 0–0.3; red = P: 0.3–0.6; and yellow = P: 1.0; iteration number represents the migration time. The degradation is accelerated without the protection of the heterojunction layer. (**b**) The simulation results of ion migration at room temperature when the heterojunction layer is applied on top of the perovskite film of 23% doping in the X site after iterations 1, 50, 100, 250, 500, and 1000. (**c**) The simulation results of ion migration at higher temperature in the perovskite film of 10% doping in the X site after iterations 1, 50, 100, 250, 500, and 1000. Remaining perovskite lattices = 9387, 9031, 8774, 8308, 7790, and 4183. (**d**) The simulation results of ion migration at room temperature when the heterojunction layer is applied on top of the perovskite film of 10% doping in the X site after iterations 1, 50, 100, 250, 500, and 1000. Remaining perovskite lattices = 9453, 9128, 8878, 8495, 8196, and 5056.

As all the previous simulations are conducted at room temperature, while PSCs normally work at 85 °C, the migration at higher temperatures has also been investigated. The influence of temperature can be given by the Arrhenius term, which is imposed on the 10% and 23% doping film. Meanwhile, to verify the effect of the perfect WBG PSC structure on ion migration, we also constructed a 20 × 20 lattice in the center of the lattice. In this area, the PSC lattice was pure and there were no heterogeneous ions. The results showed that the PSC structure has little effect on the migration of ions and cannot hinder the diffusion of ions. As can be seen in Figure 4b, when the heterojunction layer was applied, the number of remaining perovskite lattices was 8884 after the first iteration, which gradually decayed to 8329, 7933, 7288, 6866, and 1788 after the 50th, 100th, 250th, 500th, and 1000th iteration, respectively. The number of remaining lattices when no heterojunction layer is applied is 8094, 7539, 6601, 6063, 989, and 0, respectively (Figure 4a). The decay rate is much faster than that at room temperature conditions, especially when no heterojunction layer is applied; all perovskite lattices are destroyed in this case. Figure 4c,d demonstrate the degradation process of the perovskite film of 10% doping under the same conditions with Figure 4a,b, which exhibited a similar degradation trend with more perovskite lattices remaining as a result of smaller migration driving force. Moisture attack could also deteriorate the PSC’s stability, yet related details have been extensively investigated and well understood [18]. It has now been accepted that the addition of an extra heterojunction layer will be able to passivate the surface imperfection and hinder the water degradation, as proved in previous literature [10,17]. Therefore, our results proved the significance of applying a heterojunction layer for achieving stable WBG PSCs and tandem devices in the real working environment.

## 3. Methods

Following our previous work [32,33,34,35], the simulation was conducted by Python 3.7.0 [18], where the WBG perovskite film is demonstrated by a 100 × 100 lattice, representing the smallest repeating unit of the perovskite structure. The initial distribution of impurity atoms was randomly set to simulate actual doping. The driving force for migration alters for various doping degrees, which will further affect the perovskite’s structural stability. The average distance between all atoms may differ in various impurity atom concentrations, yet thermodynamic energies are considered as more directly related factor that affect the structural stability at both microscopic and macroscopic levels. When one lattice collapses, it opens a pathway for the ion to migrate.

In this study, two widely employed WBG perovskites were adopted [36]: MAPb(I_0.77_Br_0.23_)_3_ and MAPb(I_0.9_Br_0.1_)_3_. The doping agent and heterojunction layer are PbBr_2_ and KMnF_3_, respectively. Although the chemical nature of the dopant may vary, the driving force for ion migration can be obtained from the literature, which directly influences the perovskite structural stability. Thus, the purpose of this research is to focus on thermodynamic and kinetic energy, and investigate how those energy terms will affect the structural breakdown. The all-inorganic perovskite KMnF_3_ were introduced atop the WBG perovskite layer, which can be achieved by physical vapor depositions (e.g., sputtering). Such materials exhibit ionic bonds, providing greatly enhanced halide bonding [12]. A simplified WBG PSC configuration without a charge transport layer is applied [37], such as transparent conductive glass/WBG perovskite/transparent electrode, where ion migration is refined only within the WBG perovskite. For perovskite-Si, optical-coupled four-terminal tandem solar cells were employed and simulated for further lifetime evaluation.

In order to simulate the thermodynamics and kinetics of the migration, the following energy terms were introduced in each lattice. G represents the driving force for migration, which originated from lattice mismatch, doping degree, and concentration gradient. The number for this energy ranges from 1.3 to 1.6 eV [38]. B, which is given by the bonding energy of the perovskite structure, is at the scale of 0.5 eV [14]. E, which is the activation energy for migration, ranges from 0.4–0.6 eV [39]. The final barrier Ea* for migration could now be calculated by G-B-E. The migration probability P could be extrapolated by the exponential of the Ea* divided by the gas constant and temperature. Various colors were assigned to identify the probability P; blue implied the probability of migration was 0, meaning that the perovskite structure remained intact. Green represents 30%, yellow for 60%, and red for 100%.

To accommodate for unconventional diffusion, as the host lattice changed due to structural degradation, which was experimentally observed, it was incorporated into the simulation axioms. Instead of the usual Fickian equations connecting all lattices with the same diffusion rate, the connections are now specified as variables (W) and will be updated; each lattice point is associated with a migration probability P(x, y) and is connected to its left, right, above, and below by possible paths, denoted by W(x, y, d = 1–4), whose values will be updated during the actual ion diffusion process. The P value of a given point is calculated using the master formula, which combines the P and W values of its four neighbors. The formula shows the relationship between all these values and how they affect the updated P value of the lattice. The P value of a lattice (x, y) can be represented by Pi, whose value is shown in Equation (1).

*dP_i_*/*dt* = Σ*j*(*P_j_W_ji_* − *P_i_W_ij_*) (1)

Next, the W(x, y, d) values are updated as the migration proceeds; when any P(x, y) value reaches 0.3, its W(left) or W(right) value has a 50/50 chance of becoming 1.0, or 100% probability, simulating partial breakdown of the perovskite; and when this P(x, y) value increases to 0.6, its W(right) or W(left) will also become 1.0, leaving only the W(up) and W(down) values intact. Based on the simulation principle, various situations were simulated, including the effect of doping, multi-layer heterojunction, and high temperature. It should be noted that although the perovskite lattice parameters will affect its structural stability, such variations can be indicated by the energy terms. As has been simulated, all the energy terms that would support/destroy the structure were assigned to each lattice. Based on the influence of ion migration on the bonding energy, updates will be made to these terms after each iteration.

## 4. Conclusions

This research revealed the specific ion migration process, and the detailed thermodynamics and kinetics involved via simulation. Unlike moisture diffusion, the simulation results showed an unconventional diffusion process for ion migration, as the total amount of ions in the film is limited and will be homogenized at the end of the iteration. The migration driving force may not necessarily increase with the concentration of doped ions, as the halogen bond will hinder the diffusion, which needs to exceed a critical stage to trigger accelerated diffusion. Based on the discovery, we proposed a heterojunction layer to provide additional bonding energy to resist ion migration. As a result, the migration speed was successfully reduced by more than 100%, especially at a higher temperature. The bonding energy provided by the heterojunction later plays an important role in inhibiting ion migration. Hopefully, these findings could provide fresh inspiration for the WBG PSC research and remove the obstacles to their commercialization.

While the current study primarily focuses on numerical simulations to reveal the migration process, we expect to conduct experimental investigations and evaluate the practical stability of WBG perovskite in the follow-up study. In addition, further optimization of the structure and optoelectronic properties of heterojunction layers is also required to enhance the resultant device performance and lifetime. Moreover, with the evolvement of new-charge transport materials, we anticipate that stable-charge transport layers will be integrated into WBG PSCs and tandem devices to further enhance their lifetime.

## Data Availability

The original contributions presented in the study are included in the article, further inquiries can be directed to the corresponding author/s.
